# Continuous Re-MTAPA Block via SEDIC Catheter Placement: A Novel Analgesia Technique in Two Complex Surgical Cases

**DOI:** 10.7759/cureus.84087

**Published:** 2025-05-14

**Authors:** Keisuke Nakazawa, Tasuku Watari, Rei Ikeda, Takahiro Suzuki

**Affiliations:** 1 Anesthesiology, Nihon University School of Medicine, Tokyo, JPN

**Keywords:** alternative to epidural, catheter technique, fascial plane block, multimodal analgesia, postoperative analgesia, regional anesthesia, re-mtapa, sedic, thoracoabdominal nerves, ultrasound-guided regional anesthesia

## Abstract

Epidural anesthesia is commonly used for perioperative pain management, but it can be challenging in patients with anatomical issues or those on anticoagulation therapy. We present two cases in which catheter placement in the space between the endothoracic fascia, diaphragm, and costodiaphragmatic recess (SEDIC) was used for postoperative analgesia. This technique was applied to two patients: one with severe thoracolumbar scoliosis (American Society of Anesthesiologists Physical Status (ASA-PS) 2) undergoing laparoscopic distal gastrectomy and another receiving antiplatelet therapy (ASA-PS 3) undergoing open abdominal aortic aneurysm repair. A continuous infusion of 0.125% levobupivacaine, supplemented with twice-daily boluses of 10 mL of 0.25% levobupivacaine via bilateral catheters, was administered. Both patients maintained Numeric Rating Scale scores below 3 throughout the observation period and did not require rescue analgesia. Continuous re-modified thoracoabdominal nerves through perichondrial approach block via SEDIC catheter placement provided effective postoperative analgesia in patients with contraindications to epidural techniques, supporting early mobilization without hemodynamic instability.

## Introduction

Epidural anesthesia remains the gold standard for perioperative pain management in major abdominal surgeries. However, it is often contraindicated in patients with spinal deformities or those receiving anticoagulant or antiplatelet therapy. Additionally, epidural anesthesia is associated with notable side effects, including arterial hypotension in up to 20% of patients, urinary retention in 15-30% of cases, and motor blockade, which can delay early mobilization [1]. These complications may hinder postoperative recovery.

The development of fascial plane blocks has introduced alternative analgesic options. Traditional blocks such as the rectus sheath block (RSB) and the transversus abdominis plane block (TAPB) primarily target the anterior cutaneous branches of the intercostal nerves. However, studies have shown that TAPB can produce inconsistent and variable sensory block patterns, with significant individual differences [2].

The thoracoabdominal nerves through perichondrial approach (TAPA) block, first described by Tulgar et al. in 2019 [3], is a novel technique that affects both anterior and lateral branches of the thoracoabdominal nerves. In the original TAPA method, local anesthetic is administered to both the lower and upper surfaces of the chondrium at the costochondral corner. This approach provides sensory coverage from the T5 to T12 dermatomes, making it suitable for thoracic, breast, and abdominal procedures [3].

Shortly thereafter, Tulgar et al. introduced the modified TAPA (M-TAPA) technique, in which the anesthetic is delivered only to the lower surface of the chondrium [4]. More recently, Ohgoshi et al. described the re-modified TAPA (Re-MTAPA) technique, which targets a newly identified anatomical space known as the space between the endothoracic fascia, diaphragm, and costodiaphragmatic recess (SEDIC) [4]. This approach effectively blocks both the anterior and lateral cutaneous branches. Notably, Ohgoshi et al. observed that “the SEDIC and M-TAPA plane are spatially completely separated by either costal cartilage or a tendinous structure,” indicating that local anesthetic administered via the standard M-TAPA is unlikely to diffuse into the SEDIC space spontaneously [5].

While TAP blocks mainly affect the anterior branches, resulting in central abdominal sensory loss [2], the Re-MTAPA technique may offer more comprehensive analgesia by also targeting the lateral cutaneous branches [5].

Here, we report two cases in which continuous Re-MTAPA block via SEDIC catheter placement provided effective postoperative analgesia in patients for whom epidural anesthesia was either technically challenging or relatively contraindicated.

This case was presented as a poster at the 12th Annual Meeting of the Japanese Society of Regional Anesthesia (JSRA) on April 18, 2025.

## Case presentation

Anesthetic management

**Figure 1:**
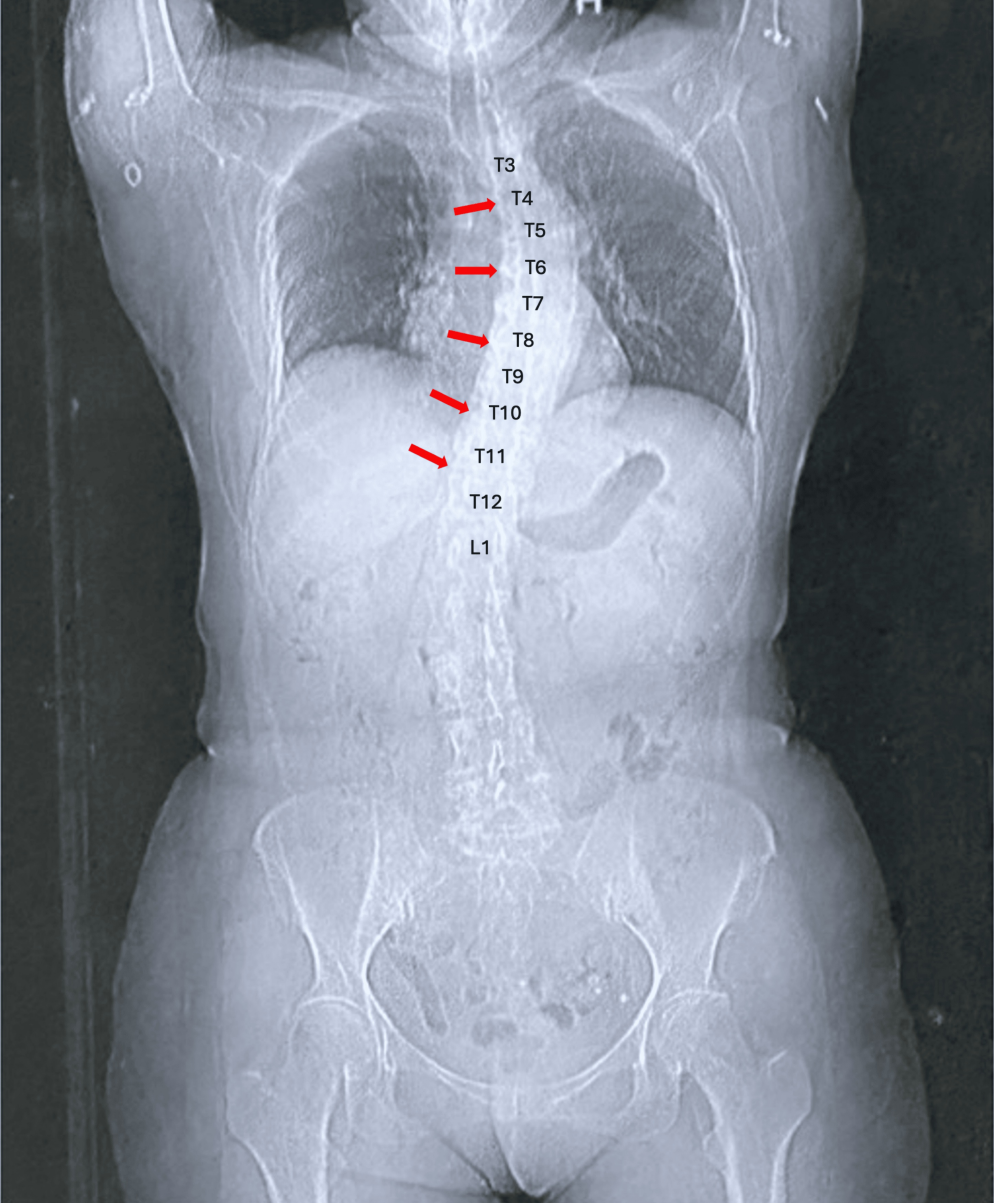
Case 1: Anteroposterior radiograph showing thoracic scoliosis This image reveals marked curvature of the mid-thoracic spine (T7-T11), the typical level for epidural catheter placement in abdominal surgeries. Red arrows indicate the direction of scoliosis, and increased white radiographic density is observed at the compressed vertebral bodies. These anatomical changes posed technical difficulties for epidural access, prompting the use of the SEDIC catheter technique for the Re-MTAPA block. L, lumbar vertebrae; T, thoracic vertebrae

Two patients with contraindications to epidural anesthesia were selected for this novel analgesic approach. The first patient had significant thoracolumbar scoliosis, which rendered epidural catheter placement technically challenging (Figure 1). The second patient was on continuous antiplatelet therapy, presenting a relative contraindication to neuraxial techniques.

For Case 1, standard monitoring was established, including invasive arterial pressure monitoring. General anesthesia was induced and maintained with fentanyl, remifentanil, propofol (target-controlled infusion at 2.0-3.0 μg/mL, targeting a bispectral index (BIS) of 40-60), and rocuronium (0.6 mg/kg for induction, with intermittent 10 mg boluses to maintain a train-of-four count of 0). Remifentanil was maintained at 0.25-0.40 μg/kg/min. IV fentanyl 200 μg was administered one hour before the end of surgery. Acetaminophen 675 mg (15 mg/kg) was infused slowly over 15 minutes at the start of skin closure.

For Case 2, standard monitoring included invasive arterial pressure, central venous pressure, BIS, and neuromuscular monitoring. Total IV anesthesia was delivered using remimazolam (0.1 mg/kg bolus followed by a continuous infusion at 0.5-1.0 mg/kg/hr), rocuronium (0.6 mg/kg for induction, with 10 mg intermittent boluses), and remifentanil (starting at 0.4 μg/kg/min, adjusted to 0.25-0.40 μg/kg/min after intubation). IV fentanyl 500 μg was administered one hour before the end of surgery. Acetaminophen 1,000 mg was infused slowly over 15 minutes during skin closure.

SEDIC catheter technique

Upon completion of the surgical procedures and while the patients remained under general anesthesia, bilateral Re-MTAPA blocks were performed via SEDIC catheter placement using an identical technique for both cases. Patients were placed in the supine position for the block.

A linear ultrasound probe was positioned parallel to the ninth/10th costal arch to visualize key anatomical structures: the external oblique muscle, intercostal muscles (ICM), diaphragm, and transversus abdominis muscle (TAM).

On ultrasound imaging, a distinct histological boundary between the TAM and the diaphragm was clearly visible. This boundary corresponds to the tendinous structure previously described by Ohgoshi et al. [4]. The SEDIC was identified as the area located cranially, between the deep surface of the ICM and the diaphragm, above this tendinous structure.

**Figure 2:**
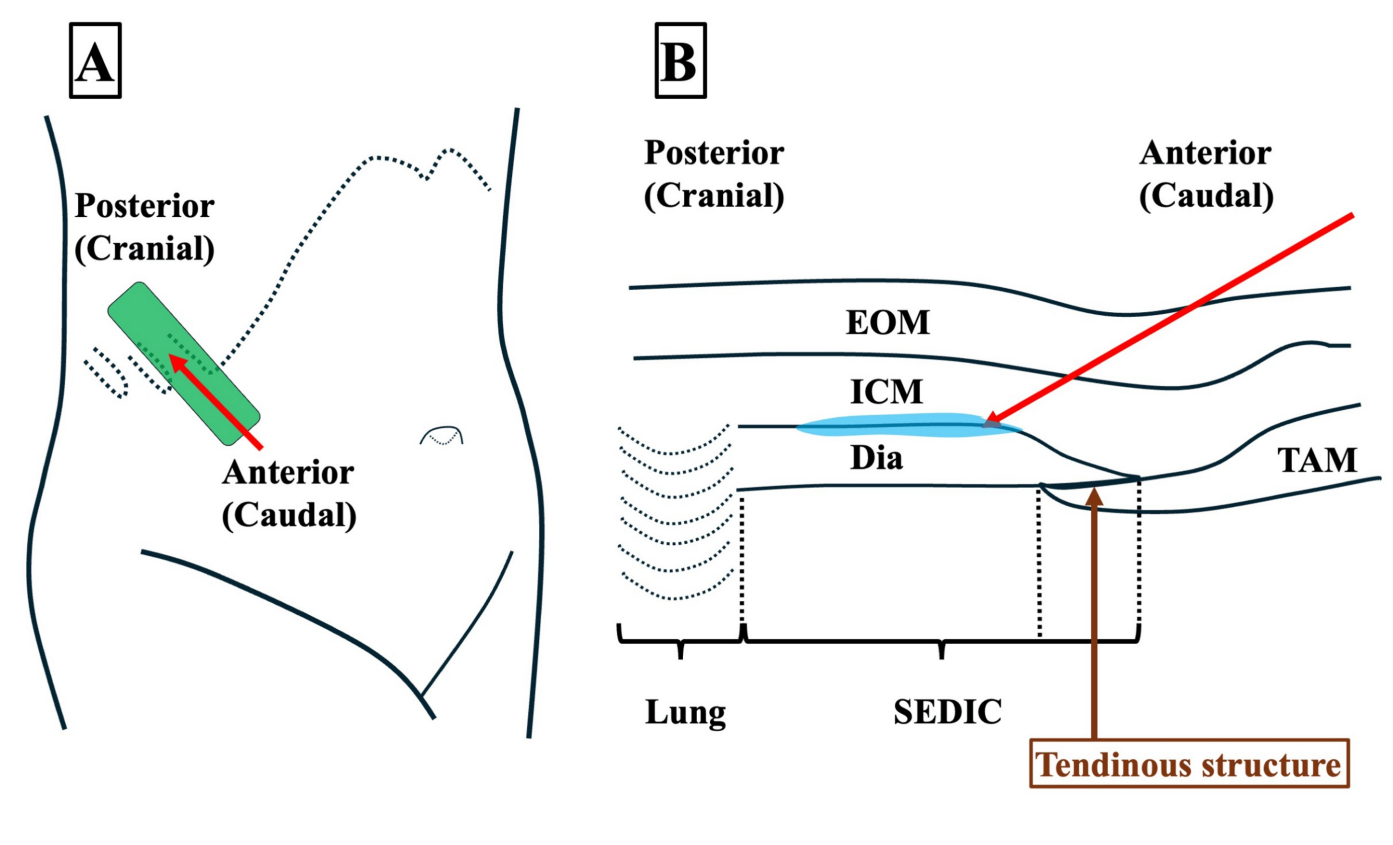
SEDIC space identification and needle approach for Re-MTAPA block (A) Probe positioning for the Re-MTAPA block. A linear ultrasound probe (green rectangle) is placed parallel to the intercostal space at the T9-T10 costal arch level, oriented to avoid visualization of the costal cartilage. The red arrow indicates the planned needle trajectory. (B) Corresponding ultrasound image showing key anatomical structures. The SEDIC space is delineated inferiorly by the tendinous structure at the transition between the Dia and TAM, and superiorly by the acoustic shadow cast by the lung. The red arrow shows the needle path, inserted at a 30-45° angle relative to the probe surface. After penetrating the fascial layer of the ICM just cranial to the tendinous structure, 5 mL of saline (blue color) is injected to confirm hydrodissection between the ICM and diaphragm. Upon confirmation of correct plane entry, local anesthetic is administered to visualize its spread within the SEDIC space. A catheter is then advanced 5 cm beyond the needle tip and secured to the trunk. All procedures used a Contiplex® Touhy Ultra360^®^ needle (18G, 100 mm). EOM, external oblique muscle; Dia, diaphragm; ICM, intercostal muscles; Re-MTAPA, re-modified thoracoabdominal nerves through perichondrial approach; SEDIC, space between the endothoracic fascia, diaphragm, and costodiaphragmatic recess; TAM, transversus abdominis muscle

Figure 2 illustrates the ultrasound probe positioning and the resulting image used to identify the SEDIC space, highlighting the key anatomical landmarks and the needle trajectory for the Re-MTAPA block.

**Figure 3:**
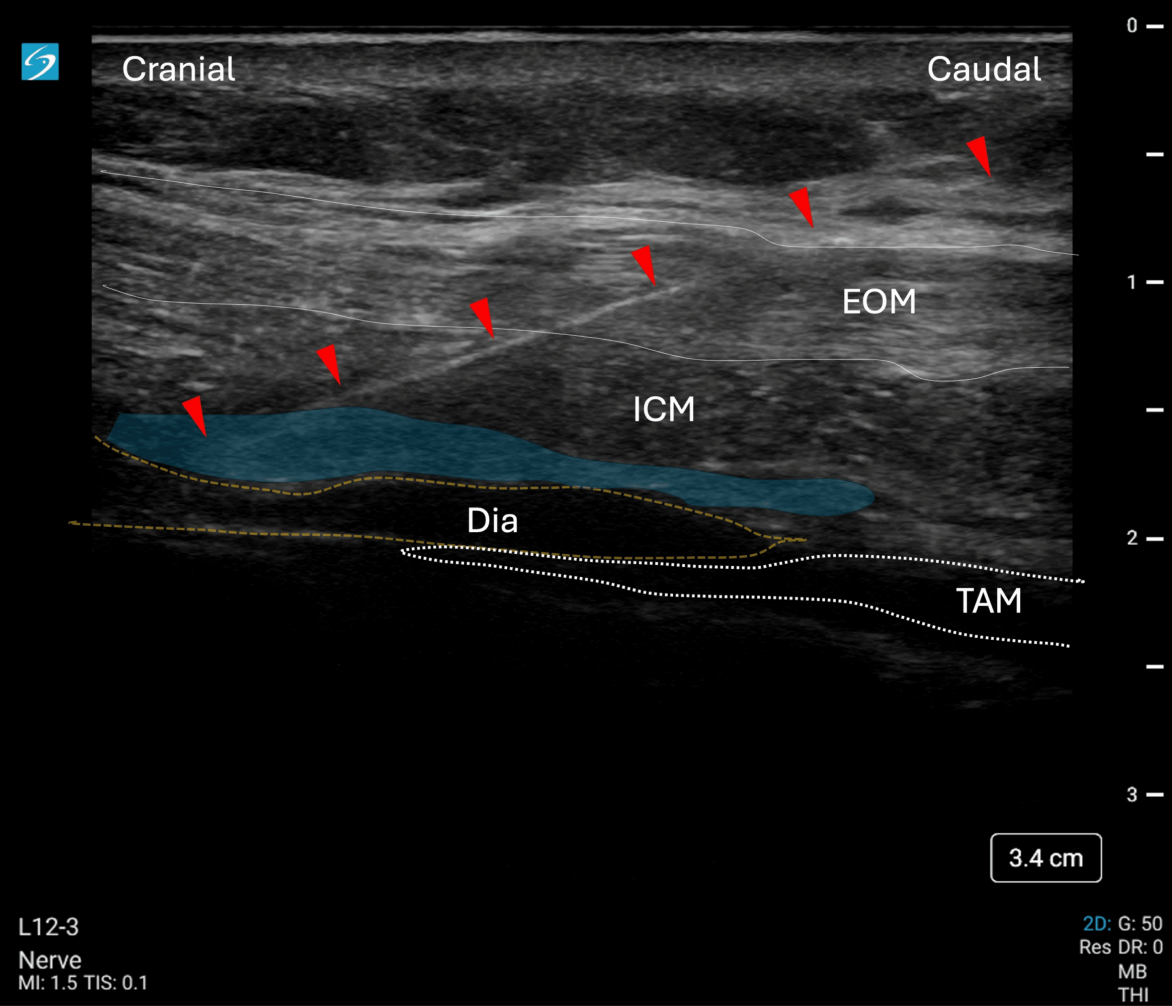
Case 1: Ultrasonographic visualization of local anesthetic injection into the SEDIC space during Re-MTAPA block The image displays the ultrasound-guided technique using a linear probe aligned parallel to the 9th/10th costal arch. Key anatomical structures are labeled: EOM, ICM, Dia, and TAM. The blue-highlighted area indicates the spread of local anesthetic within the SEDIC. Red arrowheads mark the needle trajectory from caudal to cranial. EOM, external oblique muscle; Dia, diaphragm; ICM, intercostal muscles; Re-MTAPA, re-modified thoracoabdominal nerves through perichondrial approach; SEDIC, space between the endothoracic fascia, diaphragm, and costodiaphragmatic recess; TAM, transversus abdominis muscle

Using an in-plane approach, a Contiplex^®^ Touhy Ultra360^®^ (18G, 100 mm) needle was advanced from the caudal to the cranial direction at a 30-45° angle relative to the probe surface. After penetrating the fascial layer of the ICM on the cranial side of the tendinous structure, hydrodissection with 5 mL of saline was performed to enhance visualization of the anatomical compartment prior to local anesthetic injection. This step was essential to confirm accurate needle placement between the ICM and the diaphragm within the SEDIC space. The spread of the local anesthetic is highlighted in blue in Figure 3.

For Case 1, 20 mL of 0.25% levobupivacaine was administered per side (total 40 mL), and for Case 2, 30 mL per side (total 60 mL), with total doses kept below the maximum recommended limit of 2.5 mg/kg to reduce the risk of local anesthetic systemic toxicity (LAST). Following confirmation, a catheter was advanced 5 cm beyond the needle tip and secured with Steri-Strips and Tegaderm dressing. Sensory assessment was performed 15 minutes after each bolus injection using both cold testing with alcohol-soaked gauze and a pinprick test.

Postoperative analgesia protocol

A standardized multimodal analgesia regimen was implemented, comprising a continuous infusion of 0.125% levobupivacaine at 4 mL/hour through each catheter, supplemented with scheduled boluses of 0.25% levobupivacaine (10 mL per catheter) administered at 7:00 AM and 6:00 PM. Additionally, IV patient-controlled analgesia (IV-PCA) was provided, consisting of fentanyl delivered at a continuous rate of 15 μg/hour, with 15 μg bolus doses available and a 10-minute lockout interval. For breakthrough pain (defined as Numeric Rating Scale (NRS) ≥5), rescue analgesia options included oral acetaminophen (600 mg) or loxoprofen (60 mg). Pain assessments were performed three times daily - at 7:00 AM, 1:00 PM, and 6:00 PM - by nursing staff or members of the anesthesiology team, with NRS scores documented both at rest and during movement. Monitoring continued until catheter removal in both cases.

Case 1

A 68-year-old woman (height: 155 cm; weight: 45 kg) with severe thoracolumbar scoliosis was scheduled for laparoscopic distal gastrectomy. Due to the pronounced lateral curvature of the spine and significant vertebral rotation, epidural catheter placement was not feasible. The deviation of the spinal column from the midline posed an anatomical challenge that necessitated an alternative approach to analgesia.

The surgical procedure lasted three hours, and the total anesthesia time was four hours and 43 minutes. Upon completion of the laparoscopic surgery, bilateral Re-MTAPA block via SEDIC catheter placement was performed as described earlier.

**Figure 4:**
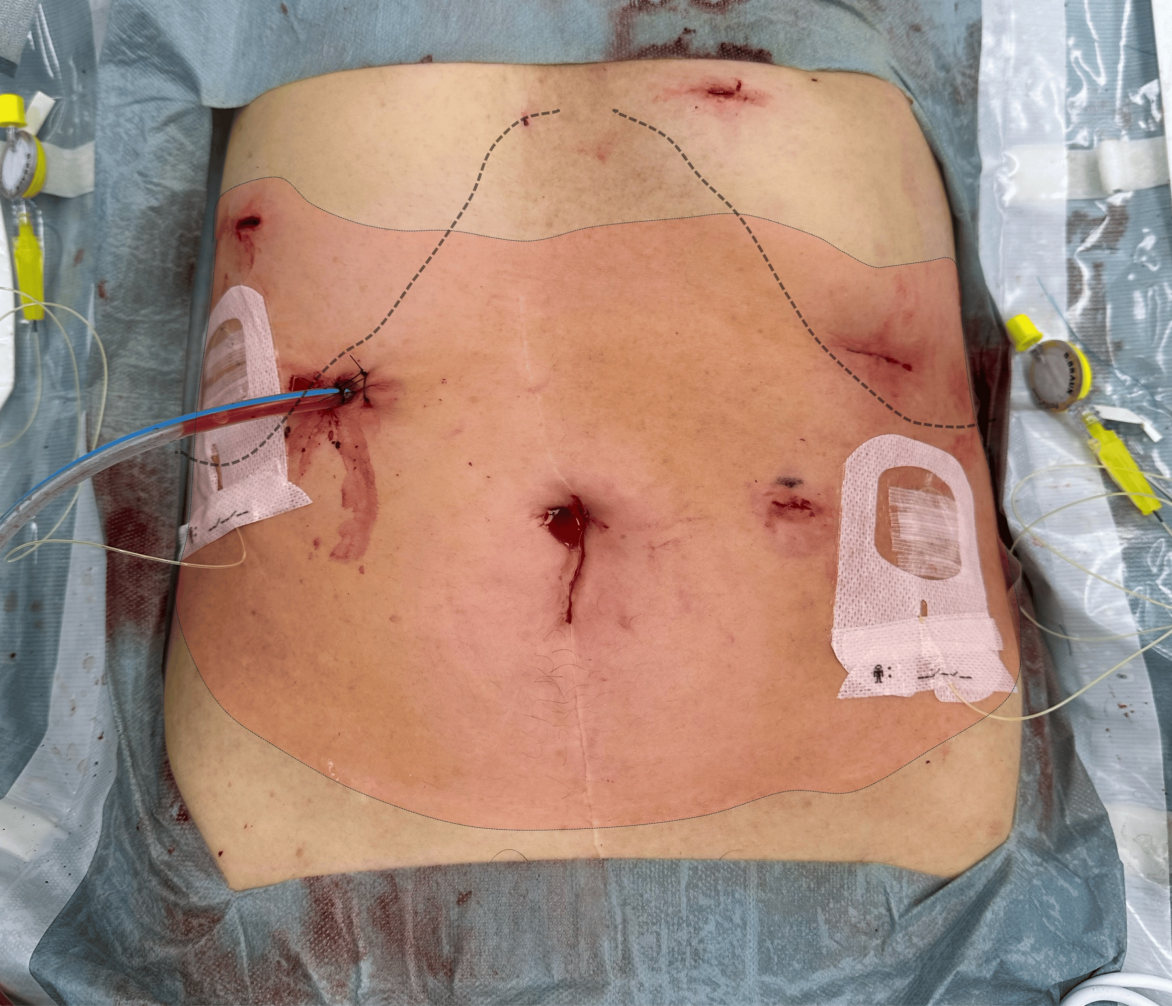
Case 1: Sensory assessment following SEDIC local anesthetic administration The outlined area represents the region of diminished sensory perception assessed using both the cold test (with alcohol-soaked gauze) and the pinprick test, performed 15 minutes after local anesthetic administration via the SEDIC catheter. The sensory blockade spans the T7-T11 dermatomes and extends laterally beyond the midclavicular line, encompassing the right hypochondrial drain site, surgical port sites, and the left hypochondrial trocar insertion site. These findings confirm effective analgesia of both anterior and lateral cutaneous branches achieved through continuous Re-MTAPA block via SEDIC catheter placement. Re-MTAPA, re-modified thoracoabdominal nerves through perichondrial approach; SEDIC, space between the endothoracic fascia, diaphragm, and costodiaphragmatic recess

Sensory assessment using both the cold test (with alcohol-soaked gauze) and pinprick test, conducted 15 minutes after bolus administration and upon emergence from general anesthesia, revealed extensive blockade of the T7-T11 dermatomes. The sensory loss extended laterally beyond the midclavicular line. The area of diminished cold sensation corresponded with the location of the right hypochondrial drain, surgical port sites, and the left hypochondrial trocar insertion site, indicating effective blockade of both anterior and lateral cutaneous branches (Figure 4).

**Figure 5:**
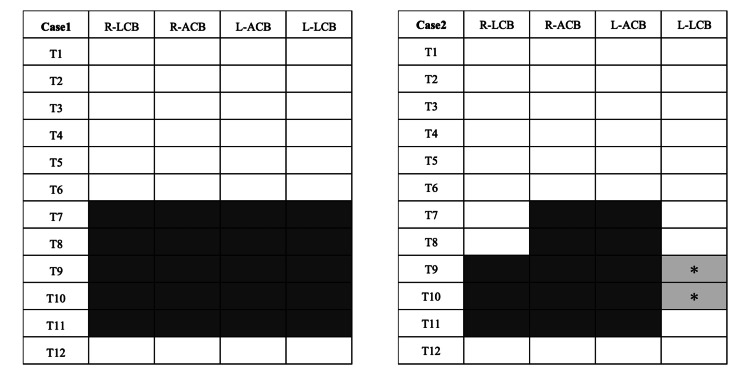
Dermatomal distribution of sensory block after Re-MTAPA block via SEDIC catheter placement This figure illustrates the extent of sensory loss, assessed using both the cold test with alcohol-soaked gauze and pinprick test, following the Re-MTAPA block via SEDIC catheter placement in both cases. Black-filled areas indicate complete sensory loss in the corresponding dermatomes, while gray areas with asterisks (*) represent regions with partial sensory loss, particularly in the lateral cutaneous branch territories where sensory changes did not extend beyond the mid-axillary line. The assessment covered both anterior cutaneous branch territories (from the midline to the mid-clavicular line) and lateral cutaneous branch territories (from the mid-clavicular line extending beyond the mid-axillary line toward the posterior axillary line). The sensory blockade was evaluated based on both cold and pinprick sensations at each testing point. L-ACB, left anterior cutaneous branch territory; L-LCB, left lateral cutaneous branch territory; R-ACB, right anterior cutaneous branch territory; R-LCB, right lateral cutaneous branch territory; Re-MTAPA, re-modified thoracoabdominal nerves through perichondrial approach; SEDIC, space between the endothoracic fascia, diaphragm, and costodiaphragmatic recess; T, thoracic intercostal nerve dermatomes

A detailed dermatomal mapping was created to systematically document the distribution of sensory blockade, demonstrating comprehensive coverage of both anterior and lateral cutaneous territories in this case (Figure 5).

Sensory assessments using both the cold test with alcohol-soaked gauze and the pinprick test were conducted after each scheduled bolus administration through the catheter (morning and evening) until catheter removal. The dermatomal distribution of the sensory block remained consistent with the initial assessment, showing a persistent and effective blockade of the T7-T11 dermatomes throughout the duration of catheter placement. Postoperative pain was well controlled throughout the recovery period.

On postoperative day 1, the patient reported NRS 0 at rest from morning until bedtime, with only mild pain (NRS 3) during rehabilitation activities and movement. This effective analgesia allowed for early mobilization, including bedside standing and bathroom ambulation. On postoperative day 2, pain control remained excellent, with NRS 0 at rest and NRS 3 during movement until catheter removal. The patient was able to complete a 200-meter hallway ambulation on day 2. No rescue medication was required during the observation period. The IV-PCA fentanyl infusion (15 μg/hour) was maintained for 48 hours postoperatively before being discontinued, with a total of 720 μg of fentanyl administered during this period. According to nursing records, no additional bolus doses of IV-PCA fentanyl were required during this time.

Case 2

A 66-year-old man (height: 165 cm; weight: 62 kg) with a history of coronary artery disease on continuous antiplatelet therapy was scheduled for open abdominal aortic aneurysm repair. Due to the need for intraoperative heparinization and ongoing antiplatelet therapy, epidural anesthesia was relatively contraindicated due to the risk of epidural hematoma.

The surgical procedure lasted four hours and four minutes, with a total anesthesia time of five hours and 46 minutes. After the procedure, bilateral Re-MTAPA block via SEDIC catheter placement was performed while the patient remained under general anesthesia.

**Figure 6:**
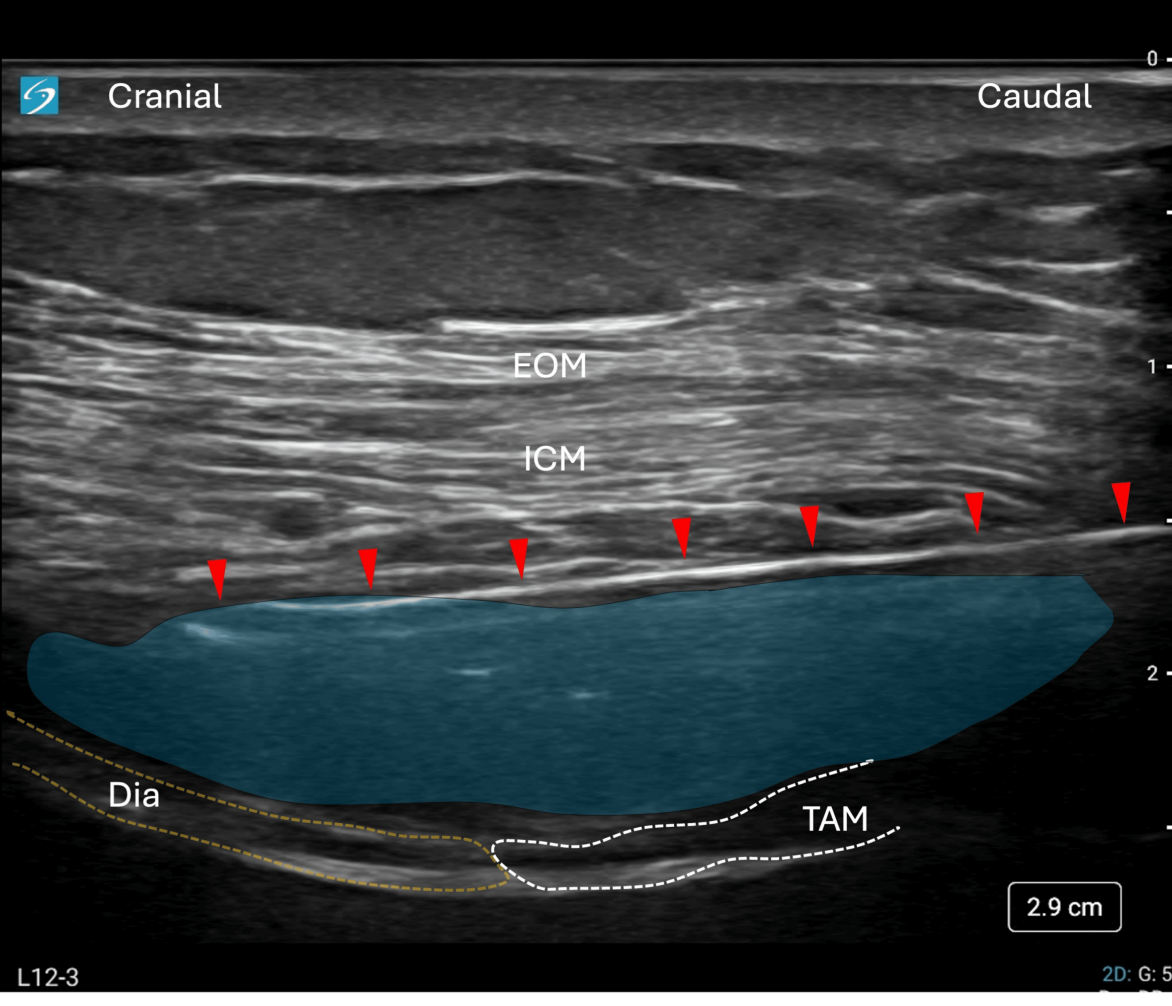
Case 2: Ultrasonographic visualization of local anesthetic injection into the SEDIC space during Re-MTAPA block The linear ultrasound probe was positioned parallel to the 9th/10th costal margin, visualizing the EOM, ICM, Dia, and TAM. The blue-highlighted area represents the spread of local anesthetic administered into the SEDIC. Red arrowheads indicate the needle trajectory. After hydrodissection, the local anesthetic demonstrated preferential spread toward the caudal transversus abdominis fascial plane. EOM, external oblique muscle; Dia, diaphragm; ICM, intercostal muscles; Re-MTAPA, re-modified thoracoabdominal nerves through perichondrial approach; SEDIC, space between the endothoracic fascia, diaphragm, and costodiaphragmatic recess; TAM, transversus abdominis muscle

We observed differences in local anesthetic spread compared to Case 1 during the ultrasound-guided technique. In Case 2, both the TAM and diaphragm were thinner, making anatomical recognition of the tendinous structure beneath the costal margin challenging on both sides. When the needle was advanced into what was believed to be the SEDIC space and local anesthetic was administered, we observed precise dissection and spread beneath the ICM. However, the local anesthetic predominantly spread toward the needle insertion site on both sides, unlike the cranial spread pattern seen in Case 1 (Figure 6).

**Figure 7:**
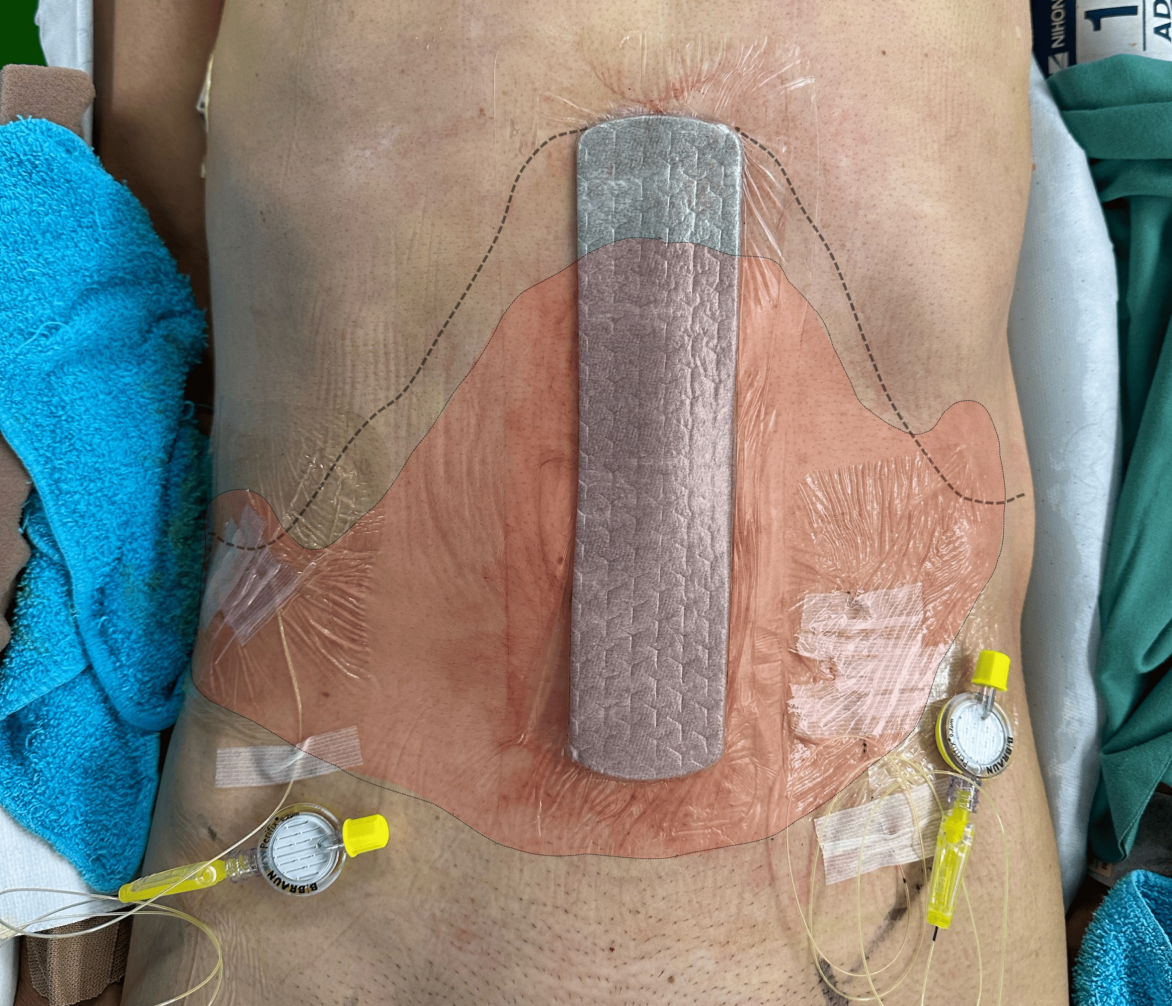
Case 2: Sensory assessment using both cold test with alcohol-soaked gauze and pinprick test, performed 15 minutes after SEDIC local anesthetic administration The outlined area demonstrates the region of diminished sensory perception as assessed by both cold test with alcohol-soaked gauze and pinprick test. The blockade effectively covered the anterior cutaneous branches, innervating the midline incision (T7-T11). In contrast, sensory loss in the lateral cutaneous branch distribution was significantly more limited compared to Case 1, affecting only the right T9-T11 dermatomes and a small area of T9-T10 dermatomes around the catheter insertion site on the left side. SEDIC, space between the endothoracic fascia, diaphragm, and costodiaphragmatic recess

Sensory assessment using both cold test with alcohol-soaked gauze and pinprick test, performed 15 minutes after bolus administration and emergence from general anesthesia, demonstrated effective blockade of the anterior cutaneous branches along the midline surgical incision (T7-T11). At the same time, sensory loss in lateral areas was more limited. The outlined area demonstrated diminished cold sensation, effectively covering the anterior cutaneous branches innervating the midline incision. In contrast, sensory loss in the lateral cutaneous branch distribution was significantly more limited compared to Case 1, affecting only the right T9-T11 dermatomes and a small area of T9-T10 dermatomes around the catheter insertion site on the left side (Figure 7).

Follow-up sensory assessments using both cold test with alcohol-soaked gauze and pinprick test after each scheduled bolus administration through the catheter confirmed that the dermatomal distribution of sensory blockade remained stable and consistent with the initial assessment until catheter removal.

Despite the more limited lateral blockade, pain control was excellent from the immediate postoperative period, with NRS 0 at rest and NRS 2 with movement on the day of surgery. On postoperative day 1, the patient reported NRS 0 at rest and NRS 2 during rehabilitation activities. This favorable analgesic profile remained stable through postoperative day 3, with NRS scores consistently at 0 at rest and 2 during rehabilitation activities until the evening after catheter removal.

Notably, no impairment of gastrointestinal motility was observed throughout the perioperative period, with the patient maintaining normal bowel function. Hemodynamics remained stable, and the patient was able to sit upright in bed on the first postoperative day and begin ambulatory rehabilitation. Throughout the entire postoperative course, the patient did not require any supplementary analgesics. The IV-PCA fentanyl infusion (15 μg/hour) was continued for 72 hours until the evening of postoperative day 3, delivering a total of 1,080 μg of fentanyl. Nursing records confirmed that no additional bolus doses were administered during this time.

## Discussion

Our case reports demonstrate the successful use of continuous Re-MTAPA block via SEDIC catheter placement for postoperative analgesia in patients with contraindications to epidural anesthesia. This technique provided effective pain relief while avoiding the complications associated with neuraxial techniques.

TAPA and M-TAPA blocks have been reported as effective in various abdominal procedures. The M-TAPA technique has shown efficacy in laparoscopic cholecystectomy, sleeve gastrectomy, gynecological laparoscopic procedures, ventral hernia repair, and pediatric nephrectomy [6-8]. A scoping review by Tanaka et al. identified successful applications in both open and laparoscopic surgeries, with significant reductions in postoperative pain scores and opioid consumption [9].

Çiftçi et al. reported their clinical experience with M-TAPA in five patients undergoing laparoscopic abdominal surgeries, including cholecystectomy, incisional hernia repair, and inguinal hernia repair [10]. They evaluated dermatomal distribution patterns with varying volumes of local anesthetic (30-40 mL total) and found adequate analgesia with sensory blockade from T6-T12, although specific dermatomal coverage varied between patients. Their findings suggest that adequate pain control can be achieved even with lower volumes than initially described in the literature, with all patients reporting NRS pain scores ≤4 during the 24-hour postoperative period [10].

Ohgoshi et al.’s research, combining cadaveric dissection and volunteer testing, demonstrated that direct administration of local anesthetic into the SEDIC is necessary to effectively block both lateral and anterior cutaneous branches of intercostal nerves. Their anatomical investigations revealed that the SEDIC and M-TAPA planes are completely spatially separated by either costal cartilage or a tendinous structure. Their volunteer study also showed that injections into the SEDIC effectively blocked lateral cutaneous branches, particularly on the right side, with significant anatomical variation and left-right asymmetry [5].

The varying efficacy observed in our two cases aligns with the anatomical variability and left-right asymmetry described by Ohgoshi et al. [5]. In their volunteer study, right-sided SEDIC injections reliably blocked lateral cutaneous branches in nine out of 10 participants, while left-sided injections showed much greater variability, with five out of nine participants experiencing minimal or no lateral branch blockade. Our dermatomal mapping (Figure 5) visually confirms this phenomenon, illustrating how the effective blockade of both anterior and lateral cutaneous branches in our first case and the more limited lateral blockade on the left side in our second case reflect the anatomical variation patterns attributed to the influence of the liver and heart on diaphragmatic positioning [5].

The limited spread observed in conventional TAP blocks has been well-documented. A study on patients undergoing elective laparoscopic cholecystectomy found that sensory blockade after subcostal TAP blocks was “mainly concentrated in the central part of the abdomen” and “covered only the region medial to the midclavicular line” [11]. This restricted spread pattern explains why standard TAP approaches often provide inadequate coverage for lateral incisions or port sites. The Re-MTAPA technique potentially overcomes this limitation by specifically targeting the SEDIC, although, as our second case demonstrates, success may vary.

An important consideration when performing fascial plane blocks is the risk of LAST. Griffiths et al. demonstrated that plasma ropivacaine concentrations after TAP blocks can approach toxic thresholds, with peak concentrations occurring 30-90 minutes post-injection [12]. They found that the mean peak plasma concentration was 2.54 μg/ml after bilateral TAP blocks using 3 mg/kg ropivacaine, with some individual values exceeding 4 μg/ml. Wada et al. reported that plasma ropivacaine concentrations after RSBs peaked later (>60 minutes) compared to TAP blocks, highlighting the importance of extended monitoring for LAST when performing fascial plane blocks [13].

The SEDIC block involves administering local anesthetic into an anatomical compartment between the endothoracic fascia and diaphragm, medial to the internal ICM. This region is highly vascularized, with proximity to intercostal vessels that could potentially lead to rapid systemic absorption of local anesthetic. Although specific studies examining plasma local anesthetic concentrations following SEDIC blocks have not yet been conducted, it is reasonable to expect rapid systemic absorption similar to or potentially higher than that observed with other fascial plane blocks and intercostal nerve blocks. In our cases, we carefully limited the levobupivacaine dose to below 2.5 mg/kg in both patients, considering the potential for rapid systemic absorption from this highly vascular area.

The intermittent bolus technique we employed (twice-daily administration of 10 ml of 0.25% levobupivacaine) may offer advantages over continuous infusion alone. This approach optimizes the spread and duration of analgesia while minimizing the total local anesthetic dose. However, careful monitoring for LAST symptoms after each bolus is essential, as peak plasma concentrations may occur at variable times following injection.

An important aspect to consider is the effect on the autonomic nervous system. While our technique avoids the sympathetic blockade commonly associated with epidural anesthesia, which can cause arterial hypotension reported in up to 20% of patients [1], the sympathetic nervous system plays an important role in transmitting visceral pain. The effective analgesia observed in our cases suggests that the somatic component of post-surgical pain was adequately controlled while avoiding the hemodynamic instability often associated with epidural analgesia.

Our approach, combining SEDIC catheter-based regional anesthesia with low-dose IV-PCA fentanyl (15 μg/hour continuous infusion), allowed us to appropriately control visceral pain components while maintaining stable hemodynamics. This beneficial profile potentially allows for earlier mobilization without the hypotension that can complicate conventional epidural approaches. The implementation of this strategy in these cases demonstrates that Re-MTAPA block via SEDIC catheter placement, combined with low-dose continuous opioid infusion, provided effective analgesia without requiring breakthrough doses. Both cases achieved reliable pain control along the midline region, confirming the technique’s effectiveness for anterior cutaneous branch blockade, regardless of variations in lateral coverage.

The 2022 European Society of Regional Anaesthesia and Pain Therapy (ESRA) guidelines on regional anesthesia in patients on antithrombotic drugs classify TAP blocks and RSBs as “superficial blocks” with low bleeding risk [14]. This classification is crucial for patients like our second case, who require continuous antiplatelet therapy and intraoperative heparinization.

In recent years, truncal fascial plane blocks have been reported as safe alternatives to epidural blocks for patients with significant comorbidities or anatomical challenges. Studies have demonstrated the safety and efficacy of RSB with monitored anesthesia care for anesthetic management of patients with severe liver cirrhosis, in whom neuraxial techniques are often contraindicated [15]. The effectiveness of the rectointercostal fascial plane block with catheter placement between the rectus sheath and ICM at the T7 costal margin has also been reported for managing upper abdominal drain site pain after cardiac surgery with cardiopulmonary bypass [16].

Additionally, the clinical utility of paraspinal fascial plane blocks, such as erector spinae plane block (ESPB) and intertransverse process (ITP) block, has been established as effective alternatives to epidural analgesia. Sia et al. conducted a meta-analysis demonstrating that ESPB significantly reduces both intraoperative and postoperative opioid requirements, with lower incidences of nausea and vomiting in laparoscopic abdominal surgeries [17]. The ITP block, performed in a slightly deeper fascial plane than ESPB, has also been reported to provide effective postoperative analgesia when used with catheter placement for abdominal surgeries [18].

Current ESRA guidelines recommend local anesthetic infiltration as the first-line approach for laparoscopic abdominal surgeries, with fascial plane blocks such as ESPB and TAP blocks recommended as second-line options for cases with severe pain [19]. This shift reflects the growing acceptance of multimodal analgesic approaches for minimally invasive procedures. The Re-MTAPA technique described in our case report offers another superficial block option targeting a newly identified myofascial space. Similar to RSB and TAP blocks, it provides clinicians with an expanded arsenal for regional anesthesia when neuraxial techniques are contraindicated or unfavorable.

To our knowledge, this is the first report of catheter placement specifically targeting the SEDIC for continuous analgesia. While Ohgoshi et al. previously reported continuous M-TAPA for major abdominal surgery [20], our approach represents a distinct advancement in technique. Unlike their method of catheter placement in the M-TAPA plane, we deliberately performed hydrodissection to identify and access the SEDIC space before catheter insertion.

Several limitations must be acknowledged. Our findings cannot be generalized to broader patient populations as a two-case report. The technique’s efficacy is likely anesthesiologist dependent, requiring precise ultrasonographic identification of the SEDIC and accurate catheter placement. Additionally, while we documented the initial dermatomal spread after bolus administration, we did not collect detailed temporal data on the duration of effect after each intermittent bolus, the progressive changes in cold sensation mapping over time, or the specific pattern of sensory recovery following catheter removal. A more comprehensive assessment of these parameters, including quantitative measurements of sensory block intensity and precise duration of analgesic effect post-catheter removal, would provide valuable insights into the technique’s pharmacodynamic profile.

Optimal local anesthetic concentrations, volumes, and administration protocols for the SEDIC approach merit further investigation. Questions remain regarding how this technique compares with other analgesic approaches in terms of efficacy, safety profiles, and patient outcomes. Given the emerging variety of truncal fascial plane blocks, determining which techniques best suit specific surgical procedures and patient populations remains a challenge. Future research may explore how individual anatomical variations influence block success and whether certain patient subgroups benefit more from this approach than others. These unresolved questions highlight the need for prospective comparative studies to better define the role of Re-MTAPA block via SEDIC catheter placement within multimodal analgesia protocols.

## Conclusions

Continuous Re-MTAPA block via SEDIC catheter placement offers a promising alternative to epidural anesthesia for patients who are contraindicated for neuraxial techniques. As shown in our novel approach, this method provides effective analgesia, facilitates early mobilization, and avoids hemodynamic instability. While the reproducibility of lateral cutaneous branch blockade effects requires further investigation, our multimodal analgesic approach - combining intermittent bolus administration through SEDIC catheters with low-dose IV-PCA fentanyl - achieved high-quality postoperative analgesia in both cases, eliminating the need for rescue analgesics. By specifically targeting local anesthetic delivery to the SEDIC rather than the conventional M-TAPA plane, our technique may offer improved blockade of both anterior and lateral cutaneous branches. Further research is necessary to optimize the technique and determine its place in perioperative pain management protocols.
